# Trends in polysubstance use in the Croatian general population: evidence from 2011-2023 national surveys

**DOI:** 10.3325/cmj.2026.67.95

**Published:** 2026-04

**Authors:** Bruno Škovrlj, Renata Glavak, Tkalić

**Affiliations:** Ivo Pilar Institute of Social Sciences, Zagreb, Croatia

## Abstract

**Aim:**

To examine the extent of polysubstance use in the general population of Croatia, determine trends in polysubstance use between 2011 and 2023, and identify distinct subgroups based on the patterns of polysubstance use and compare these subgroups across survey years.

**Methods:**

Four studies were conducted between 2011 and 2023 on nationally representative samples of Croatian residents aged 15 to 64, with a total of 19 730 respondents, through face-to-face surveys in private households. Last-year substance use (tobacco, alcohol, cannabis, ecstasy, amphetamines, cocaine, and LSD) was assessed using the Croatian translation of the European Model Questionnaire.

**Results:**

Across all four survey years, alcohol (71.8%-83.3%), tobacco (39.4%-43.7%), and cannabis (5.1%-10.2%) were the most commonly used substances in the Croatian general population. Last-year abstinence showed a declining trend between 2011 and 2023, single-substance use remained stable, and polysubstance use increased over this period. Latent class analysis identified three distinct subgroups: alcohol-only use, alcohol and tobacco use, and polysubstance use. Alcohol and tobacco use and polysubstance use classes increased in size from 2011 to 2023, with the polysubstance use class increasing from 0.9% in 2011 to 2.8% in 2023, alongside higher probabilities of cocaine and ecstasy use over time.

**Conclusion:**

Polysubstance use is a growing public health concern in Croatia. The trends of co-use of alcohol and tobacco and stimulants use with polysubstance use patterns were rising. These trends emphasize the need for further research and the development of targeted prevention and intervention efforts, accounting for distinct effects of polysubstance use and cultural specificities of Croatian society.

Polysubstance use implies the use of multiple substances within a certain time frame (1). It includes both using multiple substances on separate occasions (sequential use) and using multiple substances at the same time (concurrent or simultaneous use). Although various combinations of co-used substances have been reported, certain regularities have emerged. Alcohol, opioids, and amphetamines are typically reported as primary substances, while cannabis and cocaine are typically described as secondary substances (2).

Polysubstance use has been consistently linked to a range of adverse outcomes, including greater risk of overdose (3) and negative psychological effects (1). Individuals who use multiple substances are more likely to engage in risky behaviors (4), which elevate the risk of incarceration and sexually transmitted diseases (5), as well as mental health problems, including suicidal ideation (6). Polysubstance use has been associated with poorer treatment outcomes, higher relapse rates, and increased mortality (3,7,8).

Individuals who use substances often engage in polysubstance use. Kedia et al (9) emphasized that the vast majority of individuals who used stimulants also used other substances, primarily alcohol, tobacco, and cannabis. Connor et al (10) reported that cannabis-dependent participants who engaged in polysubstance use showed higher rates of tobacco, alcohol, and amphetamine use, stronger negative cannabis expectancies, and more severe psychiatric symptoms. Liu et al (11) found that the most common two-substance combinations among individuals receiving treatment for substance use disorders (SUDs) were cocaine and alcohol, alcohol and cannabis, and cocaine and cannabis, with the most prevalent three-substance combination involving cocaine, alcohol, and cannabis.

Empirical evidence from Croatian samples supports the widespread nature of polysubstance use. A nationally representative study showed that the highest last-year prevalence of cocaine and amphetamines use was observed among individuals who consumed alcohol on 20 or more days in the previous month. These findings were consistent in the subsample of younger adults (15-34 years) (12). Also, Glavak Tkalić et al (13) documented polysubstance use among nightclub attendees in Zagreb, including using both alcohol-illicit drug co-use and the use of multiple illicit drugs within a single night.

Several studies have documented temporal changes in polysubstance use patterns. Zuckermann et al (14) reported a significant increase in the co-use of two, three, and four substances among high school students between 2013 and 2018, with a particularly steep rise in the co-use of four substances. Dai (15), analyzing trends in alcohol, tobacco, and cannabis use from 1991 to 2017, found increasing co-use of alcohol and cannabis, alongside declining co-use of alcohol and tobacco and co-use of all three substances. Increasing trends have also been observed in clinical populations. Durand et al (16) reported rising co-detection of methadone and benzodiazepines with cocaine among patients in methadone-maintenance treatment from 2010 to 2020.

Despite accumulating evidence on the prevalence, patterns, and adverse consequences of polysubstance use, most research reporting national substance use trends continues to adopt a single-substance approach (17). Similarly, clinical studies on SUDs have mainly conceptualized these disorders as drug-specific constructs, often treating previous or current polysubstance use as an exclusion criterion (2). Such an approach may limit the understanding of the unique psychoactive effects and harms associated with polysubstance use.

This study aims to 1) examine the extent of polysubstance use in the general population in Croatia, 2) analyze trends in polysubstance use across four general population surveys conducted between 2011 and 2023, and 3) identify distinct subgroups based on the patterns of polysubstance use and compare these subgroups across survey years. This approach was adopted in response to the predominant and unjust underrepresentation of polysubstance use within the literature addressing national trends, which has largely favored a single-substance approach.

## Participants and methods

### Participants

Data were collected in four consecutive general population surveys based on representative samples of Croatian residents aged between 15 and 64 years. The surveys employed a cross-sectional design and face-to-face interviews in participants’ households. The target sample size of 5000 respondents per study was determined based on the results from previous national surveys and acceptable margins of error for substance use prevalence estimates (18-20).

### Instruments

The use of seven types of substances (tobacco, alcohol, cannabis, ecstasy, amphetamines, cocaine, and LSD) in the last year was assessed using the Croatian translation of the comprehensive European Model Questionnaire (EMQ) (21), which is commonly used in general population surveys on substance use across Europe (22). Socio-demographic variables included sex and age, which was categorized into five subgroups: 15-24, 25-34, 35-44, 45-54, and 55-64 years.

### Procedure

Data were obtained through four research projects on substance use in the general population in Croatia (18-20,22). Representative samples of Croatian residents aged 15 to 64 living in private households were drawn using the Croatian population censuses (23-25). A probabilistic multistage stratified sampling procedure was employed, with stratification by county and settlement type (urban vs rural), resulting in 42 strata. Addresses were randomly selected in proportion to the population size. Within each household, the individual with the most recent birthday was asked to participate. Data were collected using a paper-and-pencil questionnaire. Ethical approvals were granted by the Ivo Pilar Institute Ethics Committee (2011, 2015) or by the Croatian Public Health Institute Ethics Committee (2019, 2023). Participation was voluntary and anonymous, and all respondents provided informed consent. Parental or guardian consent was additionally obtained for underage participants, following the Croatian Psychological Chamber’s ethical guidelines.

### Statistical analysis

To minimize potential sampling bias and ensure representativeness in terms of birth cohort and sex within the Croatian population, post-stratification weights were applied based on age and sex distribution (22). Weights were calculated separately for each survey year by comparing the distribution of the sample with population data, thereby improving representativeness. They were applied in all analyses except for basic sociodemographic descriptions. Binary logistic regression was conducted to examine trends in polysubstance use, with the reference year adjusted to allow comparisons between successive survey years. Odds ratios and corresponding 95% confidence intervals were calculated for these comparisons. Latent class analysis (LCA) was conducted to identify subgroups within the Croatian general population based on last-year substance use. Models with varying numbers of classes were estimated separately for each survey year using maximum likelihood with multiple random starts. Model selection was based on the Akaike information criterion (AIC), Bayesian information criterion (BIC), entropy, and class interpretability (26). To examine the comparability of class structure across survey years, a multi-group LCA was conducted with survey year as the grouping variable.

## Results

A total of 19 730 respondents participated in four studies: 4756 in 2011, 4992 in 2015, 4994 in 2019, and 4988 in 2023. Response rates were 53.1% (2011), 56.2% (2015), 52.9% (2019), and 71.2% (2023). Basic sociodemographic characteristics of participants are presented in Table 1.

**Table 1:** Basic sociodemographic characteristics of participants across survey years

	2011	2015	2019	2023
**Sex (%)**				
male	44.2	45.4	42.3	43.5
female	55.8	54.6	57.7	56.5
**Age (%)**				
15-24	20.0	18.0	19.1	16.2
25-34	22.5	23.0	20.6	24.1
35-44	17.1	18.7	17.2	20.0
45-54	18.7	20.4	18.7	17.3
55-64	21.8	19.8	24.4	22.4

Across all four survey years, the most commonly used substance was alcohol, with 71.8% to 83.3% of participants reporting last-year use, depending on the survey year. A substantial proportion of the population also reported last-year use of tobacco (39.4%-43.7%) and cannabis (5.1%-10.2%). Although less prevalent, the last-year use of other illicit drugs, including cocaine (0.4%-2.7%), amphetamines (0.7%-1.8%), ecstasy (0.3%-1.2%), and LSD (0.2%-0.4%), remains a significant public health concern. The proportions of participants reporting the use of a different number of substances in the last year were calculated separately for each survey year. Table 2 shows the comparison of the prevalence across survey years.

**Table 2:** The proportion of participants reporting polysubstance use in last year, by the number of substances and survey year

Number of substances used in the last year (%)	2011	2015	2019	2023
No use	21.5	19.4	12.1	17.5
One substance	44.6	45.9	46.6	42.6
Two substances	29.7	28.2	32.5	32.2
Three substances	3.5	5.1	6.3	5.2
Four or more substances	0.8	1.3	2.5	2.5

Across all survey years, the largest proportion of participants reported using one substance in the last year (42.6%-46.6%), primarily alcohol. Results of the binary logistic regression are presented in Table 3. The number of participants who reported no substance use in the last year significantly declined across survey years, with the exception of an increase between 2019 and 2023. This decline corresponded with an increase in the proportion of participants who used at least one substance. Single-substance use remained largely stable across survey years, with a significant decline observed only from 2019 to 2023. All measures of polysubstance use significantly increased from 2011 to 2023. The largest effect size was observed for the use of four or more substances in the last year, which increased from 0.8% in 2011 to 2.5% in 2023 (OR 3.193, 95% CI 2.18- 4.68, *P* < 0.001). Dual substance use increased between 2015 and 2019, while three- and four-or-more-substance use increased from 2011 to 2015, as well as from 2015 to 2019. Three-substance use declined slightly from 2019 to 2023, but the overall trend across the entire study period was upward for all polysubstance categories.

**Table 3:** Binary logistic regression results for last-year polysubstance use across survey years*

	Odds ratio (95% confidence interval)			
	2011-2015	2015-2019	2019-2023	2011-2023
No use	0.880 (0.794, 0.974)	0.569 (0.508, 0.639)	1.548 (1.377, 1.740)	0.776 (0.699, 0.861)
One substance	1.054 (0.971, 1.145)	1.031 (0.950, 1.119)	0.850 (0.783, 0.923)	0.923 (0.850, 1.003)
Two substances	0.932 (0.852, 1.021)	1.227 (1.122, 1.340)	0.984 (0.902, 1.074)	1.125 (1.029, 1.230)
Three substances	1.512 (1.229, 1.860)	1.241 (1.041, 1.481)	0.818 (0.686, 0.976)	1.535 (1.249, 1.888)
Four or more substances	1.694 (1.115, 2.574)	1.880 (1.374, 2.571)	1.002 (0.769, 1.305)	3.193 (2.177, 4.682)

*In each comparison between survey years, the earlier survey year was used as a reference point (eg, in 2011-2015 comparison, 2011 is a reference point).

Across all survey years, based on a combination of fit indices (AIC and BIC), entropy, and interpretability of the classes, a three-class solution provided the best fit (2011: AIC = 13 511.05, BIC = 13 658.87; 2015: AIC = 14 637.00, BIC = 14 785.41; 2019: AIC = 15 033.93, BIC = 15 182.92; 2023: AIC = 15 625.05, BIC = 15 774.16), with moderate class separation with some overlap between classes (entropy: 0.643 to 0.685). Models with additional classes did not show substantial improvements in model fit or classification quality. Multi-group LCA results suggested that the measurement-invariant (restricted) model fit was significantly worse than the non-invariant (unrestricted) model (χ^2^ (63) = 272.97, *P* < 0.001), providing evidence against measurement invariance. Although the invariant model yielded a lower BIC, the unrestricted model demonstrated superior overall fit (loglik = −29,311.63, AIC = 58 807.25, BIC = 59 529.15) compared with the restricted model (loglik = −29,448.11, AIC = 58 954.23, BIC = 59 181.78), and was retained, which suggests that the indicator-class relationships varied across survey years.

Based on last-year substance use patterns, the largest identified class was the alcohol-only use class, comprising 70.2% of participants in 2011, 72.1% in 2015, 66.7% in 2019, and 60.1% in 2023. This class was characterized by a moderate to high probability of alcohol use, low to negligible probability of tobacco use, and near-zero probabilities of illicit drug use. The alcohol and tobacco use class encompassed 28.9% (2011), 26.1% (2015), 30.5% (2019), and 37.1% (2023) of participants, with high probabilities of alcohol and tobacco use, and low to zero probabilities of illicit drug use. The smallest class, the polysubstance use class, included 0.9% (2011), 1.8% (2015), 2.8% (2019), and 2.8% (2023) of participants, with high probabilities of alcohol, tobacco, and cannabis use; moderate probabilities of ecstasy, amphetamine, and cocaine use; and low probabilities of LSD use. Conditional probabilities for tobacco, alcohol, and illicit drug use across survey years are presented in Table 4.

**Table 4:** Conditional probabilities of substance use by latent class across survey years

	Alcohol-only use	Alcohol and tobacco use	Polysubstance use
**2011**			
Tobacco	0.21	0.80	0.96
Alcohol	0.60	0.96	1
Cannabis	0.00	0.14	1
Ecstasy	0.00	0.00	0.22
Amphetamines	0.00	0.00	0.69
Cocaine	0.00	0.00	0.37
LSD	0.00	0.00	0.18
**2015**			
Tobacco	0.25	0.79	0.88
Alcohol	0.64	0.98	0.99
Cannabis	0.00	0.23	0.98
Ecstasy	0.00	0.00	0.32
Amphetamines	0.00	0.00	0.53
Cocaine	0.00	0.00	0.37
LSD	0.00	0.00	0.11
**2019**			
Tobacco	0.24	0.80	0.91
Alcohol	0.75	0.98	0.98
Cannabis	0.00	0.24	0.95
Ecstasy	0.00	0.00	0.40
Amphetamines	0.00	0.00	0.59
Cocaine	0.00	0.00	0.62
LSD	0.00	0.00	0.10
**2023**			
Tobacco	0.18	0.77	0.90
Alcohol	0.64	0.98	0.98
Cannabis	0.01	0.16	0.87
Ecstasy	0.00	0.00	0.36
Amphetamines	0.00	0.00	0.45
Cocaine	0.00	0.02	0.72
LSD	0.00	0.00	0.14

Differences in both class membership prevalence and item-response probabilities across survey years warrant consideration. The alcohol-only use class increased slightly from 2011 to 2015, then gradually declined to 2023. The alcohol and tobacco use class decreased modestly from 2011 to 2015, followed by increases in subsequent years. The polysubstance use class consistently increased across the survey years, ultimately tripling in relative size from 2011 to 2023. Item-response probabilities also changed across survey years. In the alcohol-only use class, probabilities remained largely stable, apart from a slight increase in alcohol use probability in 2019. In the alcohol and tobacco use class, item-response probabilities remained stable. In the polysubstance use class, variability was observed for ecstasy, amphetamine, and cocaine use. The probability of ecstasy use increased from 2011 to 2019, and then slightly declined in 2023. Amphetamine use probability decreased in 2015, increased in 2019, and declined again in 2023. The largest change was observed for cocaine use probability, which doubled from 2011 to 2023.

## Discussion

Across the four survey years, alcohol, tobacco, and cannabis were the most commonly used substances in the Croatian general population, consistent with previous research (17). In each survey year, the largest proportion of participants reported the use of a single substance in the last year. Polysubstance use was prevalent in the general population and increased gradually across survey years. These findings align with the literature, highlighting a significant presence of polysubstance use among people who use psychoactive substances (1,9,13). Trend analysis indicated a decline in the proportion of participants reporting no substance use in the last year between 2011 and 2023, alongside an increase in all measures of polysubstance use. The most pronounced increase was observed for the use of four or more substances, which tripled from 2011 to 2023. These trends are consistent with prior studies documenting rising polysubstance use, including a particularly steep rise in the co-use of four substances among adolescents (14), and demonstrate that such trends extend into the general adult population.

LCA identified three distinct subgroups in the general population based on their last-year prevalence of alcohol, tobacco, and illicit drug use: 1) alcohol-only use; 2) alcohol and tobacco use; and 3) polysubstance use. The polysubstance use class was relatively small, particularly in 2011, which may raise concerns regarding the stability and robustness of the class structure. However, the three-class structure was consistently identified across survey years, as well as in prior research (6,27), and allowed for meaningful interpretation of substance use patterns, thus supporting its retention. More complex models (1), identifying different patterns of multiple illicit drug use, have often relied on online or convenience samples that overrepresent people who use psychoactive substances, which may account for differences in class structure.

Changes in class membership across survey years mirrored trend analysis results. The size of the alcohol-only use class declined from 2011 to 2023, while the polysubstance use class increased, consistent with prior research (14). An increase was also observed in the alcohol and tobacco use class, which contrasts with some studies that did not identify a comparable class (27) or reported declining dual use of alcohol and tobacco (15). Such differences may reflect contextual specificities of the Croatian population, where tobacco use remains widespread, with a rising trend in the general population between 2011 and 2023 (22). Within the polysubstance use class, stimulant use probabilities changed over time. While amphetamine use probability declined, the probabilities of ecstasy and, particularly, cocaine use increased, with cocaine use probability doubling from 2011 to 2023. These patterns align with prior research (28) and national trends showing stable amphetamine use but rising ecstasy and cocaine use in Croatia over the same period (22). These population trends likely explain the divergent stimulant use probabilities in the polysubstance use class. Differences in class membership prevalences across survey years should be interpreted with caution. Given the lack of measurement invariance, latent classes may not be strictly comparable across survey years. Therefore, variations in class membership prevalences may also reflect changes in the definition of classes, and not just their relative size.

The primary strength of this study is a strong potential for generalizability of its findings due to the use of large, nationally representative samples. In contrast to most general population studies, which adopt a single-substance approach, this study examined polysubstance use, allowing the identification of substance use patterns and trends that would otherwise remain unrecognized. Despite these strengths, several limitations should be acknowledged. Cross-sectional design prohibits causal inference regarding the temporal aspects of polysubstance use. Even though the procedure followed established methodological standards (22), the participants’ responses may have been affected by social desirability bias. Furthermore, the study did not include individuals without permanent residence or those in institutions (hospitals, prisons, etc.) at the time of data collection, as data collection was conducted in private households. Underrepresentation of these groups could have biased the results. The relatively low prevalence of illicit drug and polysubstance use in nationally representative samples may have limited the identification of certain patterns of illicit drug use. Also, due to moderate entropy values and a lack of measurement invariance, the LCA results, especially those suggesting variations in size and composition of latent classes across survey years, should be interpreted with caution. Examining the temporal stability and change in the predictive value of sociodemographic and psychosocial predictors of polysubstance use could also represent an important direction for future research.

Overall, these findings indicate that polysubstance use in the Croatian population is a growing public health concern, characterized by declining abstinence and an increasing proportion of individuals using multiple substances. Rising trends of polysubstance use, including increasing ecstasy and cocaine use, point to growing complexity and elevated risk, especially in the context of stimulants-alcohol co-use. The observed divergence in stimulant use underscores the dynamic nature of polysubstance use patterns, influenced by substance availability, shifts in drug markets, and changing social norms, highlighting the importance of interdisciplinary and intersectoral responses. The persistent growth of concurrent alcohol and tobacco use reflects cultural and contextual specificities of the Croatian population, suggesting that integrated alcohol-tobacco prevention strategies may be particularly relevant in Croatia. Given the distinct characteristics and elevated risks associated with polysubstance use, including specific psychoactive effects and adverse outcomes beyond single-substance use, tailored prevention, harm reduction, and treatment approaches are warranted. Further research examining the correlates and outcomes of polysubstance use is essential to inform the development of such targeted interventions.
